# Ca Solubility in a BiFeO_3_-Based System with a Secondary Bi_2_O_3_ Phase on a Nanoscale

**DOI:** 10.1021/acs.jpcc.2c00674

**Published:** 2022-04-21

**Authors:** Ulrich Haselmann, Thomas Radlinger, Weijie Pei, Maxim N. Popov, Tobias Spitaler, Lorenz Romaner, Yurii P. Ivanov, Jian Chen, Yunbin He, Gerald Kothleitner, Zaoli Zhang

**Affiliations:** † Erich Schmid Institute of Materials Science, Austrian Academy of Sciences, 8700 Leoben, Austria; ‡ Institute for Electron Microscopy and Nanoanalysis, Graz University of Technology, 8010 Graz, Austria; § School of Materials Science & Engineering, Hubei University, 430062 Wuhan, Hubei, China; ∥ Materials Center Leoben Forschung GmbH, 8700 Leoben, Austria; ⊥ Department of Materials Science, Montanuniversität Leoben, 8700 Leoben, Austria; # Department of Materials Science & Metallurgy, University of Cambridge, Cambridge CB3 0FS, U.K.; ∇ School of Natural Sciences, Far Eastern Federal University, 690950 Vladivostok, Russia; ○ Graz Centre for Electron Microscopy, Austrian Cooperative Research, 8010 Graz, Austria; ◆ Institute of Material Physics, Montanuniversität Leoben, 8700 Leoben, Austria

## Abstract

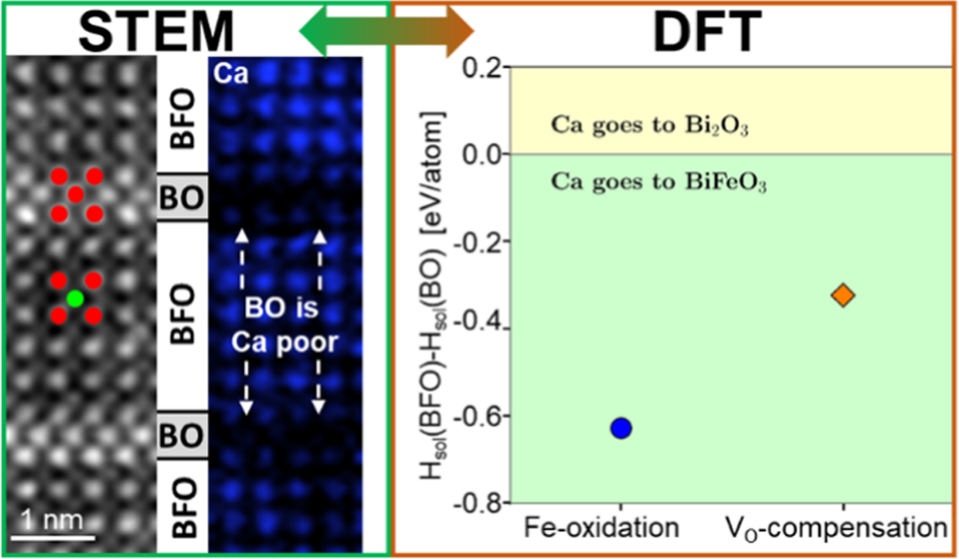

In BiFeO_3_ (BFO), Bi_2_O_3_ (BO) is a known secondary phase, which can appear under certain growth conditions. However, BO is not just an unwanted parasitic phase but can be used to create the super-tetragonal BFO phase in films on substrates, which would otherwise grow in the regular rhombohedral phase (R-phase). The super-tetragonal BFO phase has the advantage of a much larger ferroelectric polarization of 130–150 μC/cm^2^, which is around 1.5 times the value of the rhombohedral phase with 80–100 μC/cm^2^. Here, we report that the solubility of Ca, which is a common dopant of bismuth ferrite materials to tune their properties, is significantly lower in the secondary BO phase than in the observed R-phase BFO. Starting from the film growth, this leads to completely different Ca concentrations in the two phases. We show this with advanced analytical transmission electron microscopy techniques and confirm the experimental results with density functional theory (DFT) calculations. At the film’s fabrication temperature, caused by different solubilities, about 50 times higher Ca concentration is expected in the BFO phase than in the secondary one. Depending on the cooling rate after fabrication, this can further increase since a larger Ca concentration difference is expected at lower temperatures. When fabricating functional devices using Ca doping and the secondary BO phase, the difference in solubility must be considered because, depending on the ratio of the BO phase, the Ca concentration in the BFO phase can become much higher than intended. This can be critical for the intended device functionality because the Ca concentration strongly influences and modifies the BFO properties.

## 1 Introduction

Formally, multiferroics are single-phase materials with more than one of the primary ferroic properties, which are ferroelectricity, ferromagnetism, and ferroelasticity. This definition has been broadened to also include the antiferroic orders but tends to exclude ferroelasticity. The current focus of interest in the scientific community is magnetoelectric multiferroics linking electric and magnetic order parameters, which today are often only referred to by the term multiferroics.^1−4^ An important reason for this interest is a range of highly interesting possible applications. In digital data storage, the reading and writing of a magnetic bit are done by a magnetic-field-generating current that creates waste heat and has a relatively long build-up time. These disadvantages are avoided with the direct control of magnetic order via electric fields in magnetoelectric multiferroics, promising a faster, smaller, and more energy-efficient data storage.^1,5^ Other possible applications are sensors, spin valves, and spintronic devices.^6^

At the moment, BiFeO_3_ (BFO) is the leading magnetoelectric multiferroic^4^ with Curie and Neel temperatures high above room temperature (*T*_C_ ≈ 830 °C and *T*_N_ ≈ 370 °C), which is critical for device applicability.^7,8^ Multiferroic thin films often have fundamentally different properties compared to bulk materials,^9,10^ and therefore can be used to improve the properties of BFO by the choice of the substrate. Another way to tune electronic and magnetic properties is doping with substitutional elements.^11^ Ca, which substitutes Bi on the A-sites, can produce O vacancies because as an alkaline earth metal Ca^2+^ replacing Bi^3+^ causes a hole doping effect.^12,13^ Additionally, Ca doping has shown to enable modulation of conductivity through the application of an electric field,^13^ boost the magnetoelectric coupling,^14^ and shift the magnetic order from antiferromagnetic to ferromagnetic.^15^ Co, which substitutes the B-site position, leads to a significant increase in the remanent and saturation magnetization at room temperature.^16−18^ Ca and Co codoped BFO show an even larger improvement in magnetization compared to samples doped with only one of the two and are promising for obtaining a ferromagnetic system instead of an antiferromagnetic one in the future.^19^

In the BFO system, Bi_2_O_3_ (BO) is one of the secondary phases, which can form.^20−28^ BO has several times been studied in BFO thin films by TEM techniques showing a variety of different nanostructures.^29−31^ A lower substrate temperature than usual and a slower growth rate are reported to promote the formation of the secondary BO phase.^32^ The BO phase is very useful because it can be utilized to grow super-tetragonal BFO under low substrate strain conditions instead of needing a highly compressive strain, which allows growing the super-tetragonal phase on a wide variety of substrates rather than just a few suitable ones.^29,32−38^ Super-tetragonal BFO instead of its normal rhombohedral counterpart has the advantage of having a much larger ferroelectric polarization of (130–150) μC/cm^2^ instead of (80–100) μC/cm^2^.^12,39−42^

In this study, we show that the Ca solubility in R-phase BiFeO_3_ (BFO) is higher than in secondary Bi_2_O_3_ (BO), resulting in a Ca gradient between the two phases. Atomic-resolution scanning transmission electron microscopy (STEM) with high-angle annular dark-field (HAADF) imaging is used to analyze the BO nanostructure of the film and the local strain structure with geometric phase analysis (GPA). We use electron energy loss spectroscopy (EELS) and energy-dispersive X-ray spectroscopy (EDS), both in atomic resolution, to confirm the structural model and show Ca depletion in the secondary BO phase compared to the BFO-based film. This experimental result is supported by density functional theory (DFT) calculations, which show that Ca is preferentially dissolved in BFO compared to BO, and at the film growth temperature of 700 °C, about 50 times higher Ca concentration is expected in BFO than in BO. To our knowledge, this Ca solubility has not previously been reported and no pseudoternary phase diagram for Bi_2_O_3_–Fe_2_O_3_–CaO is available. Since both, Ca doping and the implementation of a BO secondary phase, are useful to tune the BFO material properties, knowledge about their interaction is crucial for the future design of BFO-based functional devices.

## 2 Experimental and Calculation Details

### 2.1 Thin-Film Fabrication

Pulsed laser deposition (PLD) with a KrF excimer laser (Lambda Physik COMPEX PRO 205 F, 248 nm) with a pulse repetition rate of 5 Hz was used to fabricate a Ca- and Co-codoped Bi_0.8_Ca_0.2_Fe_0.95_Co_0.05_O_3_ (BCFCO) and a BiFeO_3_ film on single crystalline (001)-oriented SrTiO_3_ (STO) (HeFei Crystal Technical Material Co., Ltd.) substrate. The laser beam energy per pulse was fixed at 300 mJ. The substrate was cleaned with acetone, ethanol, and pure water and afterward blown dry with high purity nitrogen gas before immediately loading it into the PLD chamber. The target–substrate distance was 5.5 cm, and the chamber was evacuated to approximately 10^–4^ Pa. During deposition, the substrate temperature was kept constant at 700 °C, and the oxygen pressure was 3 Pa. The deposition time of 10 min leads to a film thickness of 55 nm for the BCFCO film, which means a growth rate of 0.95 Å/s, and 48 nm for the BFO film, which corresponds to a growth rate of 0.80 Å/s. The crystal structure was probed in the θ–2θ scan mode with a four-circle single-crystal diffractometer (D8 discover, Bruker, Germany) using a Cu Kα_1_ monochromatic radiation source with a wavelength of 1.5406 Å. The surface topology was investigated by atomic force microscopy (AFM; Solver Nano, NT-MDT) using semi-contact mode.

### 2.2 Data Acquisition

Cross-sectional TEM samples were fabricated by standard focused ion beam protocol with a Helios Nanolab FIB-SEM.^43−46^ A probe-aberration-corrected FEI Titan^3^ operated at 300 keV at a convergence angle of 19.7 mrad was used to gain all the STEM data. For HAADF imaging, a Fischione HAADF detector was used. Detailed imaging parameters for the HAADF images can be found in Table S1. A GIF quantum^47^ energy filter (Gatan) and a super-X EDX detector^48^ (FEI) were used for the analytical data. The EELS collection semiangle was 26.6 mrad. The EELS spectra were collected in dual mode with an energy range of the core loss region of (290–802) eV and a channel width of 0.25 eV. The pixel time was 100 ms for the core loss region and 0.5 ms for the zero loss region. For the EDS maps in an energy range from 0 to 20 keV with a channel width of 5 eV, the signals from 77 images with a pixel time of 30 μs were summed up. For the EDS maps in the Supporting Information, 37 images with a pixel time of 40 μs were summed up. Drift effects were corrected during the recording with the internal Velox routine from Thermo Fisher. The differential phase contrast (DPC) data in the Supporting Information were collected using a four-segment annular FEI DF4 detector.^49^

### 2.3 Data Evaluation

For strain analysis, geometric phase analysis (GPA) software package v4.0 from HREM Research Company for Digital Micrograph 2.3 from Gatan was used. HAADF images were filtered with a principal component analysis (PCA) script for Digital Micrograph to reduce image noise written by Lichtert and Verbeeck.^50^ The EELS analytical data were processed in Digital Micrograph 3.43 from Gatan using the build-in MSA for filtering the data set.^51^ EDS data were processed with Velox from Thermo Fisher using an 8-pixel wide average filter for pre-filtering and a Gaussian with a variance of 1 for post-filtering.

### 2.4 DFT Calculations

For the ab initio calculations, a projector augmented wave (PAW)^52^ method as implemented in the VASP code was used.^53−56^ The electron exchange–correlation effects were considered using PBEsol^57^ generalized gradient approximation. The DFT + *U* method of Dudarev et al.^58^ was utilized to cope with the d-electron delocalization problem. In accordance with previous studies, for the values of the *U* parameters applied to Fe sites 4 eV was chosen.^59,60^ The energy cut-off for the basis set expansion was set to 520 eV. To compute enthalpies of solution, we employed supercells constructed from fully relaxed BFO (*R*3*c*) and BO (*P*-42_1_*c*). The BFO supercell, containing 320 atoms, was obtained by a 2 × 2 × 2 repetition of the pseudocubic unit cell. The BO supercell, containing 240 atoms, was made by a 2 × 2 × 3 repetition of the tetragonal unit cell. In addition, we considered smaller supercells to investigate higher Ca loading and found almost no difference as compared to the bigger supercells (see Figure S3). The G-type antiferromagnetic order was maintained in BFO. The Brillouin zone integration was performed using 2 × 2 × 2 *k*-point grids for the BFO and BO supercells. The positions of all atoms were relaxed until the residual forces were less than 10^–2^ eV/Å.

## 3 Results and Discussion

### 3.1 Bi_2_O_3_ Secondary Phase in the Bismuth Ferrite Matrix

**Figure 1:**
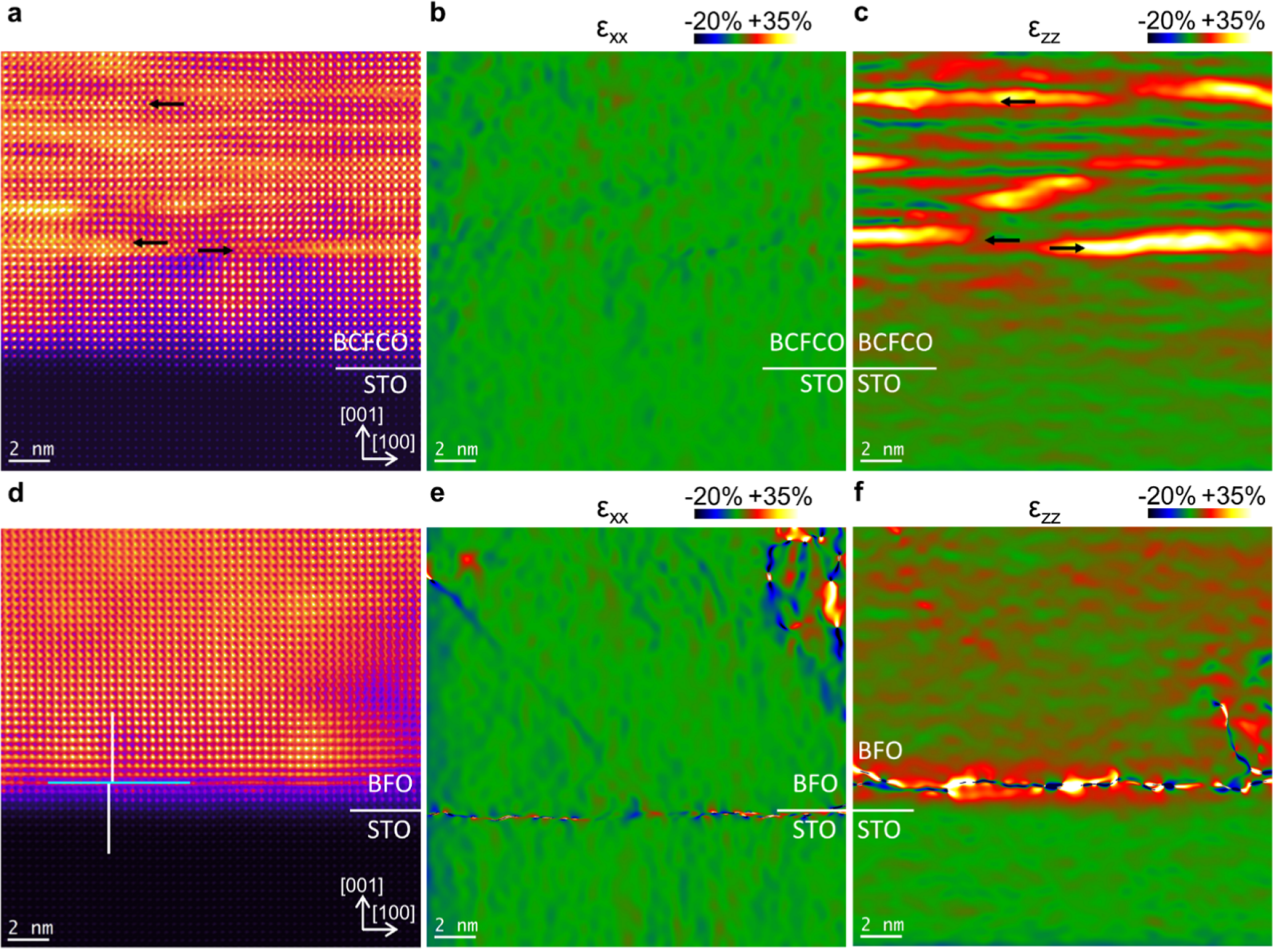
Large-scale structural mapping of a film containing the BO secondary phase and a film without it. (a) HAADF image, (b) in-plane (ε*_xx_*), and (c) out-of-plane (ε*_zz_*) strain maps of the film containing the secondary BO phase. The black arrows indicate examples of lines along the [100] direction where the B-site intensity is increased, indicating the BO phase. (d) HAADF image, (e) in-plane (ε*_xx_*), and (f) out-of-plane (ε*_zz_*) strain maps of a film containing no secondary phase. The turquoise line indicates an APB, and the adjacent white lines help guide the eyes along the A-sites to better see the shift by half a unit cell.

**Figure 2:**
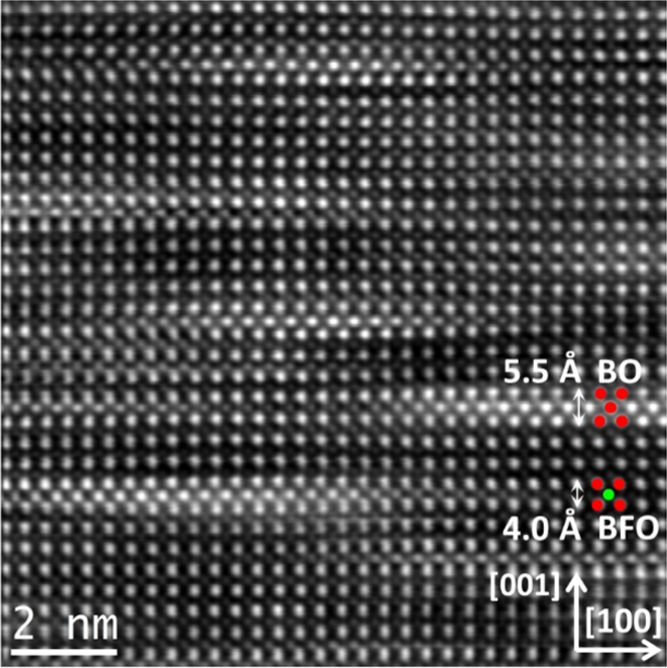
High-resolution HAADF STEM image of the secondary BO phase in the BCFCO film. The overlays indicate the majority atomic composition on the respective atomic sites (red for Bi and green for Fe).

The film structure was investigated in the [010]_c_ cross-sectional orientation concerning the cubic STO substrate, which also complies with the [010]_pc_ (pseudocubic) zone axis of bismuth ferrite.^61^ The HAADF image in Figure 1a shows a cross-section of the Ca- and Co-doped BiFeO_3_ film (BCFCO) and the interface with the STO substrate in the bottom part. In the film, approximately 5 nm away from the interface, white stripes with a width of approximately one unit cell appear running in the [100] direction. Closer inspection reveals that in these stripes, the B-site intensities are increased (the black arrows mark three exemplary examples), as is typical for a Bi_2_O_3_ (BO) secondary phase in a bismuth ferrite matrix. This is caused by the fact that on the pseudocubic lattice positions, the Fe atom on the B-site is replaced with a Bi atom, which has a much higher signal intensity in the HAADF contrast due to the higher *Z* number. The in-plane strain map in Figure 1b shows almost no strain in the film, as is expected for a good quality epitaxial growth of the film on the substrate. The out-of-plane strain map in Figure 1c unveils a relatively small lattice enlargement of the film in the first 20 nm, which is expected since the pseudocubic lattice parameter of BFO is *a*_BFO_ = 3.965 Å, which is compared to the STO *a*_BFO_ ≈ *a*_STO_ × (1 + 1.5%).^6,62^ However, apart from that, the out-of-plane strain map also shows stripes with a very large lattice enlargement, which coincides with the bright stripes in the HAADF image identified as BO and confirms their identification since the pseudocubic lattice parameter of BO is approximately 5.5 Å.^29^ For comparison, the HAADF image of an undoped BFO film on the STO substrate is shown in Figure 1d revealing no signs of the BO secondary phase. However, as indicated by the turquoise line, an antiphase boundary (APB) can be seen. The adjacent white lines are supposed to help guide the eye along the A-sites to better see the shift by half a unit cell at the APB. The in-plane strain map in Figure 1e displays some distorted areas stemming from sample damage. However, the out-of-plane strain map in Figure 1f shows, in addition to the feature from the antiphase boundary,^63^ no areas with strongly increased strain, like those we see in Figure 1c as indicators of the BO phase. A high magnification HAADF image of the BO phase inside the film is shown in Figure 2. The places where a BO phase is present can be clearly identified by the very bright B-sites, with intensities being similar to those of the A-sites. Overlays in the image illustrate the majority atomic composition on the respective atomic sites in the BO secondary phase and in the film (red indicates Bi atoms and green Fe atoms). In the [001]_pc_ direction (out-of-plane), the BO phase is usually only one unit-cell thick, and thus the BO phase seems to form plates stretching mainly in [100]_pc_ and [010]_pc_ directions within the film. As indicated in Figure 2, while the film has an out-of-plane lattice spacing of ≈4.0 Å, the BO phase has a much larger spacing of ≈5.5 Å. As some of the BO stripes end within the image area of Figure 2, the film matrix is visually distorted around the secondary phase to compensate for the much larger spacing.

### 3.2 Elemental Analysis of the Film and the Secondary Phase

**Figure 3:**
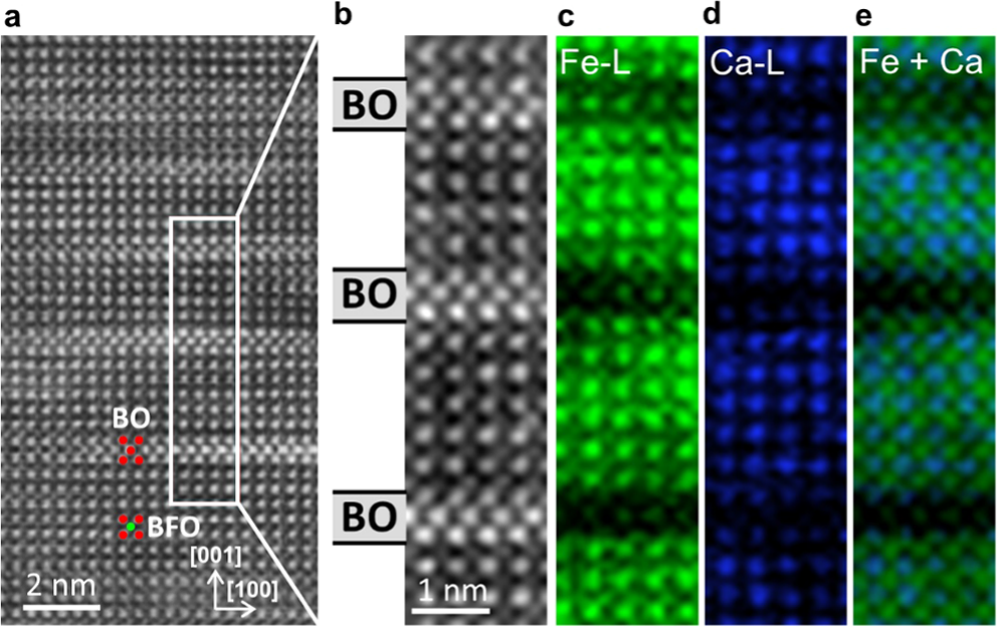
EELS elemental map of 3 plates of BO in the film. (a) HAADF survey image showing a region with several stripes of BO. The white rectangle indicates the area of the EELS elemental map containing 3 BO stripes. (b) Simultaneous HAADF image during mapping. EELS elemental maps of (c) Fe, (d) Ca, and (e) combined Fe and Ca.

Analytical STEM techniques were applied to obtain more details on the chemical composition of the film and the secondary phase. Figure 3a shows a HAADF survey image of a film region with multiple stripes of the BO secondary phase. The white, overlayed rectangle illustrates an area from which an EELS elemental map was taken. In the HAADF survey image, it clearly shows that this area contains 3 stripes of BO. Figure 3b displays the HAADF image recorded simultaneously with the EELS spectral signals. The three BO stripes oriented in the [100]_pc_ direction are clearly visible and indicated by the labeling on the left side. Figure 3c shows the Fe areal density map stemming from the Fe-L edge. As expected, in the stripes of the BO phase, the Fe content is low, with the residual Fe signal likely arising from partial intermixing in the projection direction and channeling effects. The Ca areal density map (Ca-L edge), which can be seen in Figure 3d, is very unexpected. Instead of being homogeneously distributed in the BO stripes and the surrounding matrix, which would be expected since the Ca dopant is placed in the Bi columns, the Ca content is smaller in the BO stripes than in the surrounding area. Figure 3e shows the combined elemental maps of Fe and Ca.

**Figure 4:**
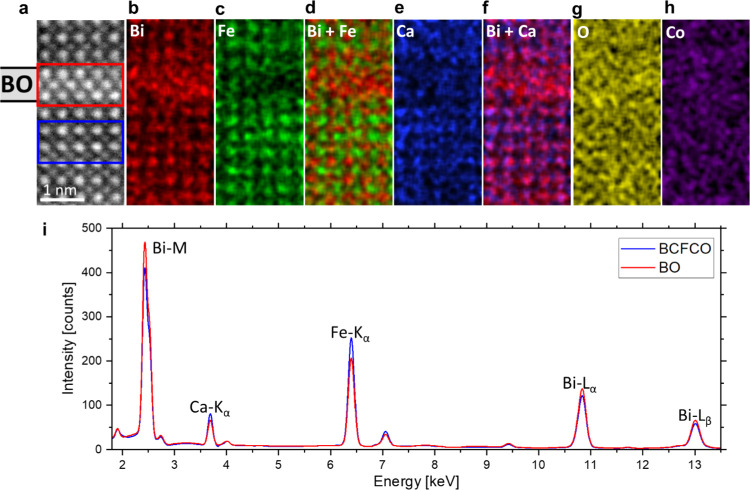
EDS elemental analysis of a BO plate. (a) HAADF image of the EDS mapping area. The BO stripe is indicated on the left side. EDS elemental ratio maps of (b) Bi, (c) Fe, (d) Bi, and Fe combined, (e) Ca, (f) Bi and Ca combined, (g) O, and (h) Co. (i) Comparing the background-corrected and denoised spectra from the BO area of the red rectangle and the BCFCO area from the blue rectangle in (a). The spectra have the same color as the associated rectangle.

To confirm the lower Ca content in the BO stripes, EDS elemental ratio maps were also recorded, which provide the advantage that Bi, which is not well suited for EELS, can be mapped too. Figure 4a displays the HAADF image of the exact area of the EDS map with a BO plate in the upper half of the image. The position of the BO plate with the clearly increased intensity of B-sites is also indicated on the left side of the image. The elemental map of Bi in Figure 4b clearly reveals a higher Bi content in the BO area, whereas the map of Fe in Figure 4c shows a lower Fe content in the BO area. In Figure 4d, the atomic ratios of Bi and Fe are combined and reveal that, while in the regular BCFCO area, there can be clearly distinguished between Bi and Fe sites, in the BO stripe the regular Fe lattice sites cannot be identified. This is also indicated by the fact that the BO stripe appears more red and less green than the regular film area, which additionally confirms the BO nature of the stripes.

Figure 4e displays the EDS map of Ca. In the area of the BO stripe, slightly less Ca can be seen. The Ca shortage in BO becomes more apparent in the combined elemental ratio map of Bi and Ca in Figure 4f. In it, the A-sites normally appear violet since red from Bi and blue from Ca color mix but in the BO stripe, it appears only red, indicating the lack of Ca. Figure 4g,h shows the O and Co maps, in which no noticeable inhomogeneities are observed. In Figure 4i, the denoised EDS spectra of BO are summed up from the area of the red rectangle in Figure 4a, and for comparison signals of the regular film are summed up from the blue rectangle. This confirms the result of a higher Bi content in the BO area and a lower Fe content indicated by different intensities of the Bi-M, Bi-Lα, Bi-Lβ, and Fe-Kα peaks. The lower Ca content in the BO stripe is confirmed by the comparison of the Ca-Kα peak, where the blue peak from the regular BCFCO film area is higher than the red peak from the BO stripe. Therefore, in addition to the confirmed BO nature of the plates, both EDS elemental analysis and EELS analysis show a lower Ca content in the BO secondary phase.

### 3.3 Solubility of Ca in Both Phases by DFT Calculations

DFT modeling was used to investigate if there is a thermodynamic driving force present that could explain the experimentally observed tendency for Ca depletion in the BO regions. To this end, the difference of enthalpies of Ca dissolution in BFO and in BO was calculated. According to our TEM analysis, Ca occupies the Bi-sites upon dissolution. This requires a charge-compensation mechanism since Ca is divalent, whereas Bi is trivalent. Two mechanisms were considered:

(1) Shift of the oxidation state of Fe from +3 to +4 (Fe-oxidation).
(2) Compensation by oxygen vacancies.

**Figure 5:**
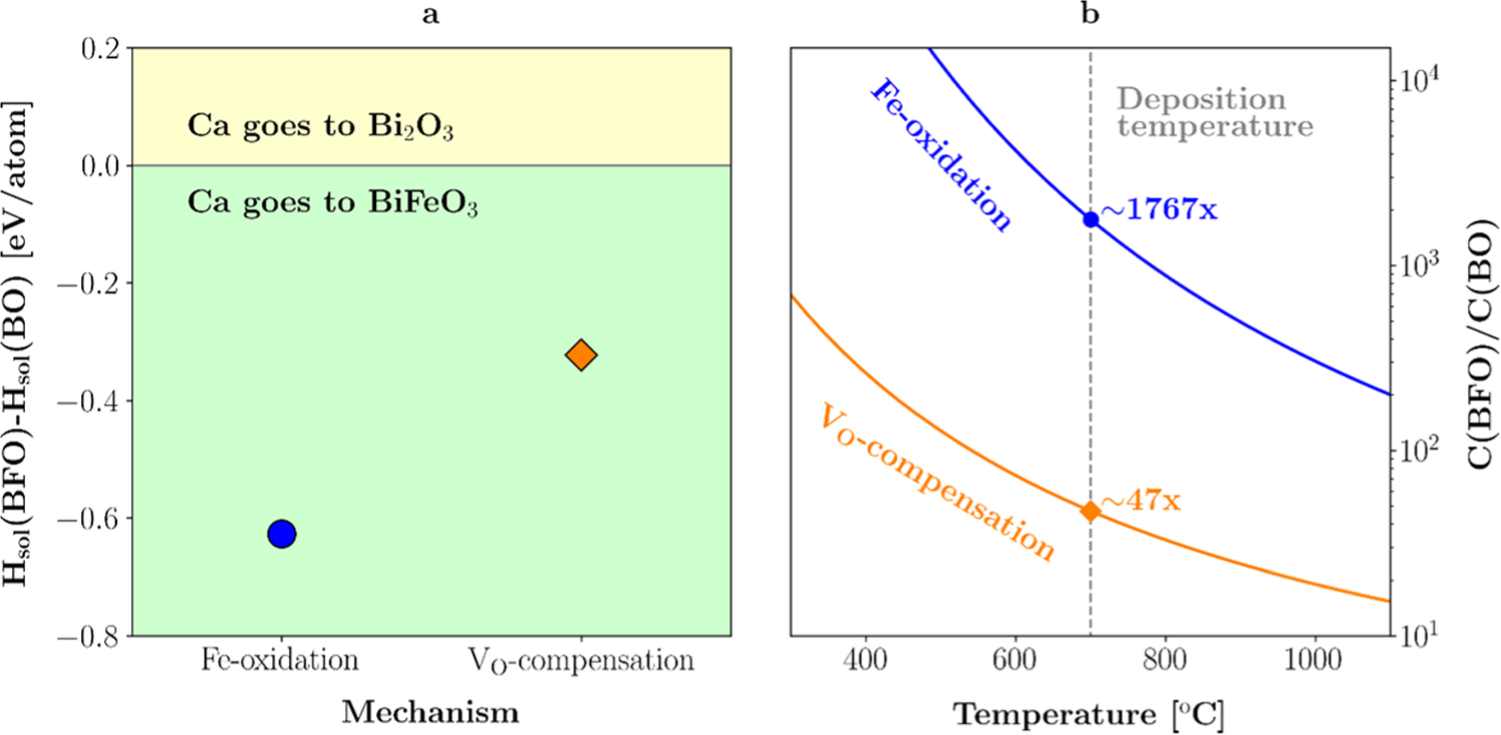
DFT calculations for the Ca solubility in BFO and secondary BO phase. (a) Relative enthalpy of Ca solution for two considered charge-compensation mechanisms: via Fe-oxidation and through the introduction of oxygen vacancies (V_O_). Both variants favor Ca dissolution in BFO. (b) Ratio of Ca concentrations for dissolution in BFO to dissolution in BO, estimated using the Arrhenius law and the data from (a). It shows that at the deposition temperature of 700 °C there is about 50 times more Ca expected in BFO than in BO.

A more detailed explanation of these mechanisms can be found elsewhere.^46,61^ The relative enthalpy of solution for the Fe-oxidation mechanism was calculated as
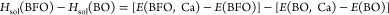
Here, *E*(BFO,Ca) and *E*(BO,Ca), are the total energies of the respective supercell containing Ca atoms, while *E*(BFO) and *E*(BO) are the energies for the supercells without Ca. The relative enthalpy of solution for the case of oxygen vacancy (V_O_) compensation was defined similarly
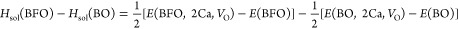
Here, *E*(BFO, 2Ca,V_O_) and *E*(BO, 2Ca,V_O_), are the total energies of the respective supercell containing two Ca atoms and one V_O_. The oxygen vacancy was always introduced in close proximity to the Ca atoms. Keeping Ca atoms and the oxygen vacancy apart was also considered and gave qualitatively the same result (see Figure S3). The relative enthalpies are presented in Figure 5a. When the relative enthalpy is negative, it means that it is energetically more favorable for the Ca to go to the BFO. If it is positive, it preferentially goes to the BO phase. The differential solution enthalpies are negative for both dissolution mechanisms, with approximately −0.32 eV for the mechanism with O vacancies and even larger (−0.63 eV) for the Fe-oxidation one. Therefore, for both mechanisms, a Ca dissolution in BFO is favored, confirming the experimental observations.

The ratio of the Ca concentration in BFO compared to the concentration in BO can also be determined from the differential solution enthalpies using the Arrhenius law
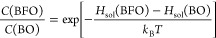
where *k*_B_ is the Boltzmann’s constant and *T* is the temperature. The result is presented in Figure 5b. The formation of O vacancies has a lower expected Ca ratio but can be considered a more likely case due to our previous results.^61^ Nonetheless, at the growth temperature of the film of 700 °C, according to the DFT simulations about 50 times more Ca is expected in BFO than in BO. With increasing temperature, the ratio becomes in general smaller. But this also means that the cooling process of the film after the fabrication step increases the Ca gradient in the secondary phase as long as the temperature still enables enough Ca diffusion.^61^

## 4 Discussion

The different Ca solubilities in the two phases observed in this study are of importance for functional device design, as the secondary BO phase leads to the super-tetragonal BFO phase under the right growth conditions, which otherwise only occurs in films grown on substrates inducing a very strong compressive strain.^29,32−38^ This allows the super-tetragonal phase to be grown on a wider range of substrates, including STO and even polycrystalline ones.^29^ In the Ca-doped thin-film sample of this study, the secondary BO phase, however, does not give rise to the super-tetragonal phase but maintains the so-called R-phase, which is monoclinic.^64^ The reasons for that could be either slightly wrong growth parameters (regarding the temperature and growth rate) or that only one unit-cell thick BO layers are too thin to induce super-tetragonal phase formation.^31^ Because the super-tetragonal phase provides a much larger ferroelectric polarization,^39,65^ it is very valuable to not be limited by the necessity of choosing a narrow range of substrates. Thereby, a relatively small change of Ca doping in the BFO phase can significantly influence and modify its properties. When using the secondary BO phase and Ca doping simultaneously, one must be aware of the Ca solubility effect because it leads, depending on the BO-phase ratio, to an increased Ca content in the BFO phase. For example, a small change of a few atomic percent Ca doping can lead to a change of the crystal structure,^13^ a switch from ferroelectric behavior to paraelectric,^13^ or a change of conductivity by up to a magnitude.^66^ This could result in a breakdown of the intended device functionality.

Previous studies have shown that related phases (Bi_2_O_2_) have some influence on the ferroelectric polarization structure by having domain walls located at the bismuth oxide layers. However, that does not mean that every bismuth oxide layer is also a domain wall. Instead, there seems to be a preference for them to be located in the bismuth oxide layers.^67^ We have also found that the BO plates can act as domain walls (see Figure S4). This could potentially be used to manipulate the location or orientation of domain walls or to influence the domain size. However, further studies are necessary for a more thorough understanding.

Note that our results also provide a highly relevant data point for assessing a thermodynamic CALPHAD database of the ternary Bi_2_O_3_–Fe_2_O_3_–CaO system. So far, only relatively little is available in this direction. Indeed, while for Bi_2_O_3_–CaO^68^ and Fe_2_O_3_–CaO^69^ systems thermodynamic models have been assessed, this is not the case for the Bi_2_O_3_–Fe_2_O_3_ system for which pseudobinary phase diagrams have been reported,^20−27^ but the correctness of the underlying experimental data is still under debate.^28^ With further progress in this direction, a thermodynamic model for the ternary Bi_2_O_3_–Fe_2_O_3_–CaO system could be in reach, where the present result provides a decisive contribution regarding Ca solubility and, in this way, also to the design of future multiferroic applications.

## 5 Conclusions

In conclusion, with advanced TEM studies and DFT calculations, we have shown that in a bismuth ferrite system with a secondary bismuth oxide phase, the Ca solubility is lower in the BO phase than in the BFO one. Different Ca solubility between the two phases can prove problematic as it increases the Ca concentration in the BFO phase and could result in a critical change of its properties. This knowledge is essential when designing functional devices. Otherwise, an unexpected Ca concentration can potentially jeopardize the functionality of the device.

## Supporting Information Available

Complementary imaging parameters of the STEM data, EELS and EDS elemental maps of the interface between the BCFCO film and the STO substrate, additional DFT calculations with higher Ca loading and alternative 2Ca + V_O_ configuration, DPC analysis of the ferroelectric polarization at a BO plate, XRD data, and AFM data of the film (PDF)
