# Supplementary material for: Ca Solubility in a BiFeO3-Based System with a Secondary Bi2O3 Phase on a Nanoscale
Source: J Phys Chem C Nanomater Interfaces. 2022 Apr 21;126(17):7696–703. doi: 10.1021/acs.jpcc.2c00674 (PMC9082603; doi:10.1021/acs.jpcc.2c00674)
Supplement: Supplementary file 1 — jp2c00674_si_001.pdf [file jp2c00674_si_001.pdf]

Supporting Information

Ca Solubility in a $BiFeO_3$ Based System with a Secondary Bi_2O_3 Phase on a Nanoscale

Ulrich Haselmann[1], Thomas Radlinger[2], Weijie Pei[3], Maxim N. Popov[4], Tobias Spitaler[5], Lorenz Romaner[4,5], Yurii P. Ivanov[6,7], Jian Chen[3], Yunbin He*[3], Gerald Kothleitner[2,8] and Zaoli Zhang*[1,9]

1 Erich Schmid Institute of Materials Science, Austrian Academy of Sciences, 8700 Leoben, Austria

2 Institute for Electron Microscopy and Nanoanalysis, Graz University of Technology,8010 Graz, Austria

3 School of Materials Science & Engineering, Hubei University, 430062 Wuhan, Hubei, China

4 Materials Center Leoben Forschung GmbH, 8700 Leoben, Austria

5 Department of Materials Science, Montanuniversität Leoben, 8700 Leoben, Austria

6 Department of Materials Science & Metallurgy, University of Cambridge, Cambridge CB3 0FS, U.K.

7 School of Natural Sciences, Far Eastern Federal University, 690950 Vladivostok, Russia

8 Graz Centre for Electron Microscopy, Austrian Cooperative Research, 8010 Graz, Austria

9 Institute of Material Physics, Montanuniversität Leoben, 8700 Leoben, Austria

*Corresponding author: zaoli.zhang@oeaw.ac.at

*Corresponding author: ybhe@hubu.edu.cn

Complementary imaging parameters of the STEM data

In Table S1 shows complementary imaging parameters of the HAADF and analytical data, which have not been mentioned in section 2. *Experimental and Calculation Details* in subsection *2.2. Data acquisition*.

Table S1. *Complementary imaging parameters of the STEM data.*

	collection angle [mrad]	pixel time [µs]	step size [pm]	scanning-frame size [pixel*pixel]
Figure 1a	49 - 242	0.95	11	2048 × 2048
Figure 1d	62 - 214	0.57	11	2048 × 2048
Figure 2	49 - 242	0.14	3.1	4096 × 4096
Figure 3a	117 - 177	11.4	65	512 × 512
Figure 3b	117 - 177	10^5	91	20 × 83
Figure 4a	124 - 200	30	17	121 × 357
Figure S1a	117 - 177	11.4	46	512 × 512
Figure S1b	117 - 177	10^5	92	26 × 72
Figure S2a	124 - 200	40	17	157 × 581
Figure S4a	39 - 200	2.5	4.24	2048 × 2048
Figure S4b	10 - 37	2.5	4.24	2048 × 2048

EELS and EDS elemental maps of the interface between the BCFCO and the STO substrate

Figure S1 and Figure S2 show elemental maps of the BCFCO-STO interface. Figure S1a shows a HAADF survey image with the area from where the spectrum images were taken indicated by the white rectangle. Figure S1b shows the HAADF image recorded simultaneously during the mapping. The areal density elemental maps Ca, Ti, Fe, and Ti combined with Fe can be seen in Figure S1c, d, e, and f. Figure S2a shows the HAADF image of the area of the EDS maps. The maps of the elemental ratios of the single elements and in some combinations can be seen in Figure S2b-j. Figure S1 and Figure S2 demonstrate that the interface between the substrate and the film is sharp and well defined.

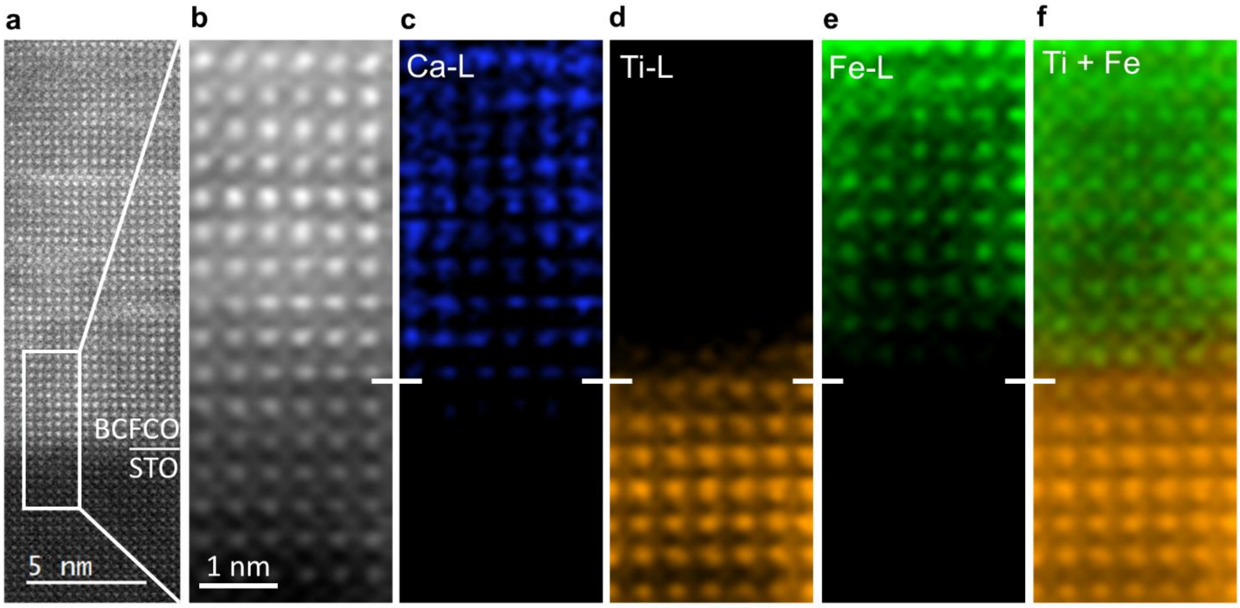

Figure S1. *EELS elemental map of the interface between the BCFCO film and the STO substrate. (a) HAADF survey image of the interface area. The white rectangle indicates the area of the EELS elemental map. (b) Simultaneous HAADF image during mapping. EELS elemental maps of (c) Ca-L, (d) Ti-L, (e) Fe-L, and (f) Ti and Fe combined. The white lines indicate the interface.*

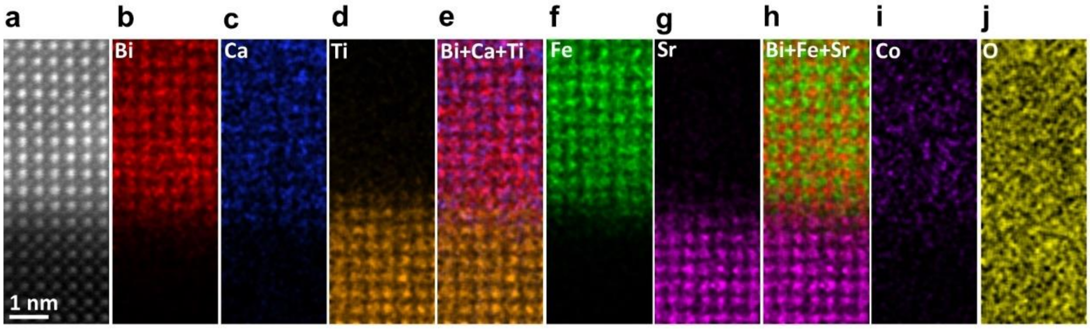

Figure S2. *EDS elemental ratio map of the interface between the BCFCO film and the STO substrate. (a) HAADF image of the EDS mapping area. EDS elemental ratio maps of (b) Bi, (c) Ca, (d) Ti, (e) Bi, Ca and Ti combined, (f) Fe, (g) Sr, (h) Bi, Fe, and Sr combined, (i) Co, and (j) O.*

DFT calculations: higher Ca loading and alternative 2 Ca + V_O configurations

In the main text, we used big supercells and computed the relative enthalpies for the dilute limit, i.e., low concentration (replacement on the Bi-sublattice with an atomic percentage of approximately 1 %). To probe the effect of higher Ca loading, we performed test calculations using unit cells instead of supercells (20 atoms in BO and 40 atoms in BFO), corresponding to Ca doping with an atomic percentage of 12.5 % on the Bi-sublattice. The result is shown in Figure S3.

It is evident, that the much higher loading of Ca (magenta and turquoise crosses) does not lead to any significant changes in the relative enthalpies.

In the main text, we put oxygen vacancy (V_O) next to the Ca ions. Our choice was dictated by a simple heuristic: both Ca ions sitting on the Bi-site and the oxygen vacancy are charged entities with opposite signs of their charges; hence, the Coulomb energy is minimized, when they sit together. Nonetheless, we performed an additional test – we put all 3 species (2 Ca ions and the oxygen vacancy) as far apart as the supercell permits. The result is also shown in Figure S3 (red diamond). Compared to the V_O being in the immediate vicinity of two Ca ions (orange diamond), we can see a reduction in the relative enthalpies. However, we should notice the following:

1) We compare two extreme cases, i.e., 2 Ca + V_O put together and as far apart as possible. That is, all other configurations are expected to fall in between the two;
2) Though the relative enthalpy is reduced, it does not change the sign, i.e., the main result of our DFT study – there is a sizeable driving force present – stays true.

Hence, we are convinced in the robustness of the DFT results presented in the main text.

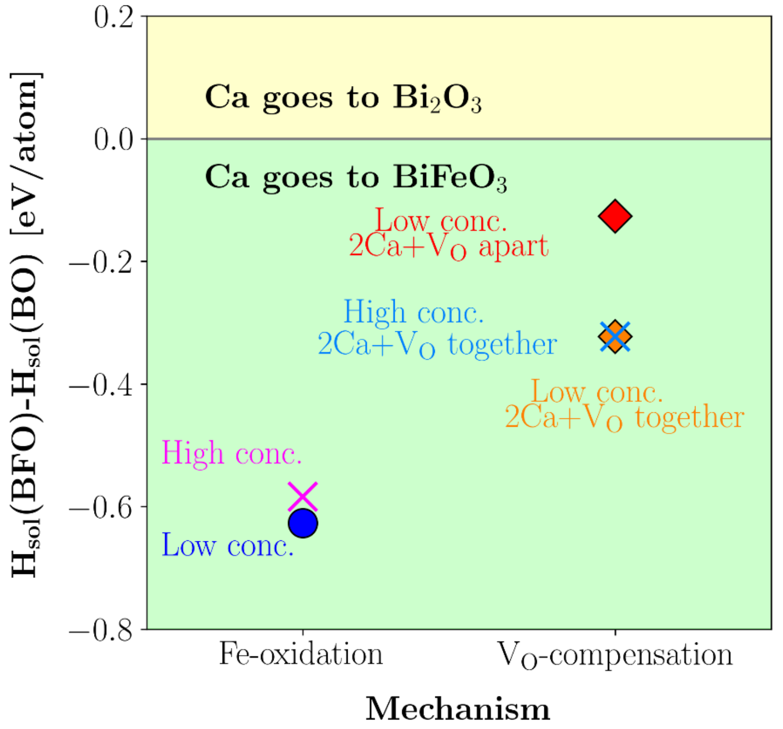

Figure S3. *Relative enthalpies of Ca dissolution in BFO and BO*

DPC analysis of the ferroelectric polarization at a BO plate

In Figure S4, the example of a BO plate acting as a domain wall can be seen. Figure S4a show the HAADF image with the location of the BO plate in the image marked on the left and right side. The BO plate divides the $Bi_{0.8}Ca_{0.2}Fe_{0.95}Mg_{0.05}O_3$ film (grown on a STO substrate) in a top and bottom area. The vector plot of the electric field[1] retrieved from the DPC signal in Figure S4b shows that in the top area above the BO plate, the ferroelectric polarization has a different orientation than in the bottom area below the plate. Therefore, the BO plate is the domain wall where the orientation changes.

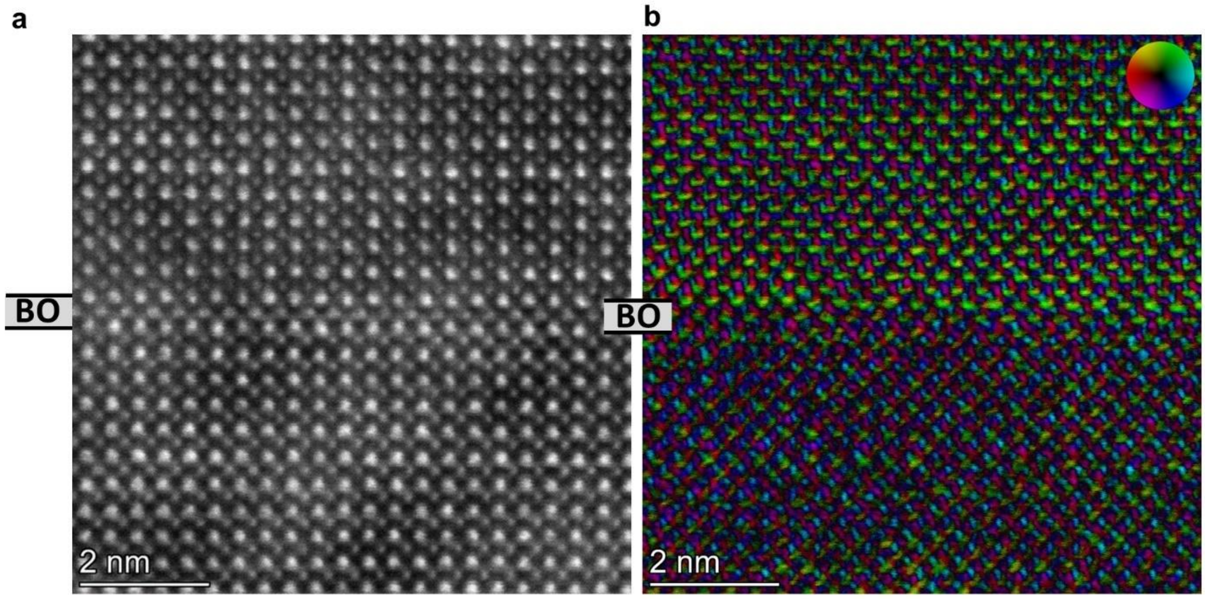

Figure S4. *DPC analysis of the ferroelectric polarization at a BO plate, which acts as a domain wall (a) HAADF STEM image of the BO plate and the area around it. The location of the BO plate is marked on the left and right side. (b) vector plot of the electric field retrieved from the DPC signal showing the two ferroelectric domains above and below the BO plate.*

XRD and AFM data of the film

Figure S5a, b show that the secondary BO phase can't be detected in the XRD data of the BCFCO film. Figure S5c shows a topographic AFM image of the BCFCO film surface with a root mean square (RMS) value of 4 nm.

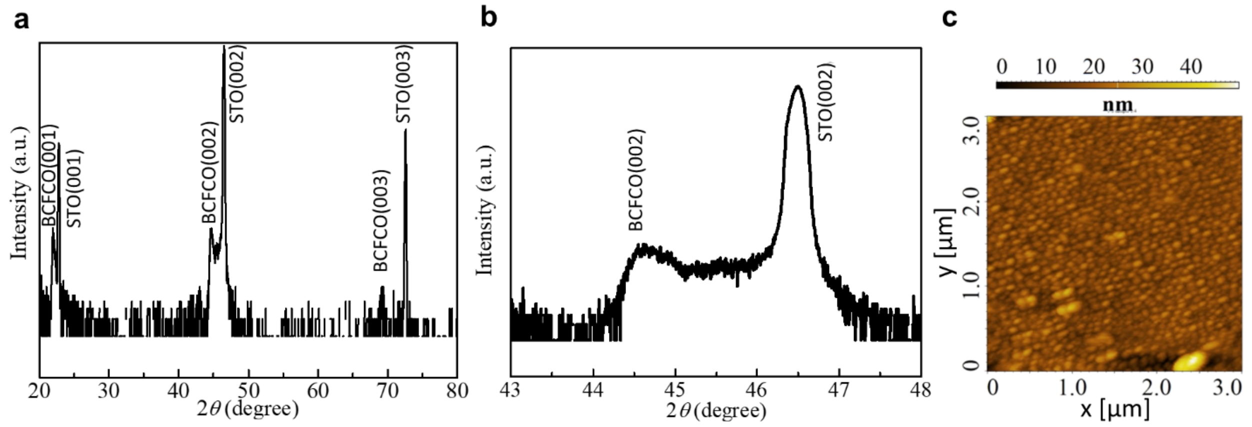

Figure S5. *(a), (b) XRD data of the BCFCO film. (c) topographic AFM image of the BCFCO film surface.*

References

(1) Campanini, M.; Erni, R.; Rossell, M. D. Probing Local Order in Multiferroics by Transmission Electron Microscopy. *Phys. Sci. Rev.* **2020**, *5*, 20190068.
